# Adenoviral E4 34K protein interacts with virus packaging components and may serve as the putative portal

**DOI:** 10.1038/s41598-017-07997-w

**Published:** 2017-08-08

**Authors:** Yadvinder S. Ahi, Ahmed O. Hassan, Sai V. Vemula, Kunpeng Li, Wen Jiang, Guang Jun Zhang, Suresh K. Mittal

**Affiliations:** 1 Department of Comparative Pathobiology, Purdue University, West Lafayette, IN USA; 2 Department of Biological Sciences, Purdue University, West Lafayette, IN USA; 3 Purdue Institute of Inflammation, Immunology, and Infectious Disease, Purdue University, West Lafayette, IN USA; 4 Purdue University Center for Cancer Research, Purdue University, West Lafayette, IN USA; 5 Merck Sharp and Dohme, West Point, PA USA; 6 HIV Dynamics and Replication Program, Center for Cancer Research, National Cancer Institute, Frederick, MD USA

## Abstract

Studies on dsDNA bacteriophages have revealed that a DNA packaging complex assembles at a special vertex called the ‘portal vertex’ and consists of a portal, a DNA packaging ATPase and other components. AdV protein IVa2 is presumed to function as a DNA packaging ATPase. However, a protein that functions as a portal is not yet identified in AdVs. To identify the AdV portal, we performed secondary structure analysis on a set of AdV proteins and compared them with the clip region of the portal proteins of bacteriophages phi29, SPP1 and T4. Our analysis revealed that the E4 34K protein of HAdV-C5 contains a region of strong similarity with the clip region of the known portal proteins. E4 34K was found to be present in empty as well as mature AdV particles. In addition, E4 34K co-immunoprecipitates and colocalizes with AdV packaging proteins. Immunogold electron microscopy demonstrated that E4 34K is located at a single site on the virus surface. Finally, tertiary structure prediction of E4 34K and its comparison with that of single subunits of Phi29, SPP1 and T4 portal proteins revealed remarkable similarity. In conclusion, our results suggest that E4 34K is the putative AdV portal protein.

## Introduction

Adenovirus (AdV) morphogenesis appears to follow a pathway similar to that observed in dsDNA containing bacteriophages and herpesviruses^1–3^. Briefly, the empty capsids are first assembled from the major capsomers, a step that possibly requires scaffolding function of an AdV protein. However, an AdV scaffolding protein is not yet identified but AdV L4 100K, pVII and pVIII seem to be important for this step^4–8^. Due to the presence of a packaging signal located close to the left end of the viral genome between the left inverted terminal repeat and the E1A transcription start site, the viral genome is specifically recognized by AdV proteins that constitute the viral packaging machinery. The AdV packaging proteins include IVa2, L4 33K, L4 22K, L1 52/55K, and possibly, DNA binding protein (DBP)^9–15^. IVa2, L4 33K and L4 22K mediate specific recognition of the viral genome^10, 16–25^. IVa2 is equivalent to the packaging ATPase proteins of dsDNA containing phages and is essential for genome packaging^9, 26^. The L4 33K, and possibly L4 22K, are required for efficient genome packaging. The L1 52/55K is likely involved in organization and stabilization of the packaged genome^27, 28^.

The process of genome packaging is well characterized for dsDNA phages such as phi29, T4, P22 and SPP1^29, 30^. The genome is packaged into precursor capsids by the action of a molecular motor that assembles at a unique vertex, the portal vertex. All known portals share a remarkable structural similarity and are invariable dodecamers of the portal protein assembled into a turbine-like structure with a central channel for the passage of genome during packaging^29, 31^. The portal is also essential for capsid assembly as it serves as the nucleation point for co-polymerization of the scaffolding protein and the capsomers^32^. Once the capsids are assembled, a complex of viral packaging ATPase associated with the viral genome and other viral proteins assembles as a ring on the portal and threads the genome into the portal channel. The genome is pushed into the capsids by the ATPase function of the ATPase protein. The ATPase activity is further potentiated by another packaging protein which binds to the ATPase.

## Results and Discussion

### Preliminary identification of putative AdV portal by secondary structure prediction

**Figure 1:**
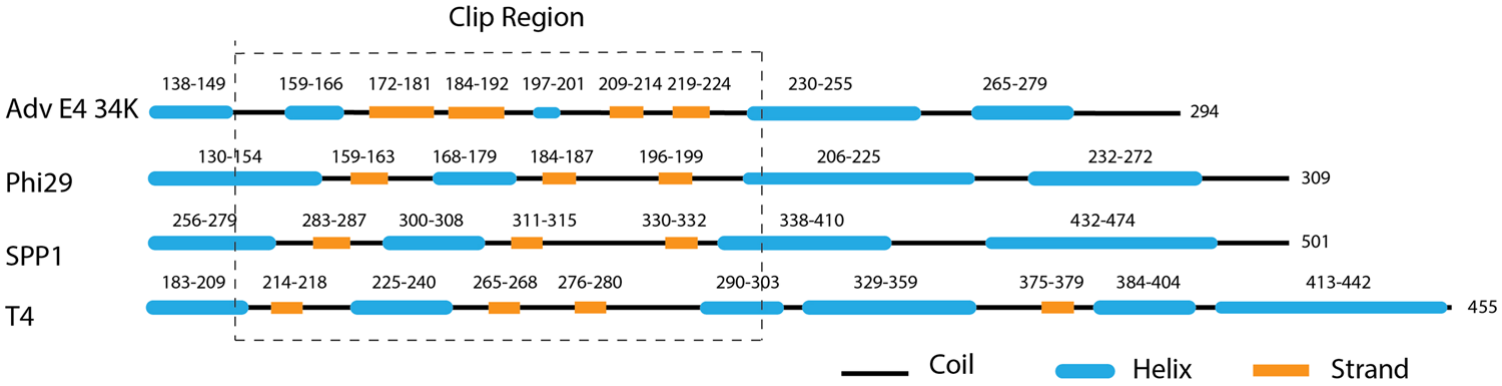
Comparison of predicted secondary structure of AdV E4 34K with the clip region of known portal proteins. The secondary structures of AdV E4 34K and three known portal proteins were predicted using PSIPRED and I-TASSER programs^40, 47^. Consensus structures are illustrated. The positions of the helices and strands are labeled above them. The predicted clip region is boxed with dash lines. The length of the predicted domain is approximately proportional to the length of the protein.

We have demonstrated earlier that IVa2, L4-33K and DBP interacts with each other and are located at a unique vertex of AdV capsids^10^. As mentioned earlier, IVa2 possibly serves as an ATPase providing energy during genome packaging. Precise roles of L4-33K and DBP during genome packaging are not clear, but L4-33K seems to potentiate ATPase activity of IVa2^9^. A structure equivalent to the portal has not been identified in AdV. Despite limited sequence similarity, all the known portal proteins share a remarkable structural similarity^33^, and as mentioned above, the portals are essential for capsid assembly^29, 31^. We compared the predicted secondary structures of a number of AdV proteins with the predicted secondary structures of known portal proteins of phages phi29, SPP1 and T4. Secondary structure analysis of E4 34K revealed a region containing a number of α -helices and β-sheets similar to the conserved fold (helix α-3 to α-6) found in the core of portal proteins and referred to as the ‘clip region’^31, 34, 35^ (Fig. 1). This observation provided the initial preliminary evidence that E4 34K may serve as the putative AdV portal. It has been suggestted that a HAdV-C5 deletion mutant lacking E4 34K and E4orf6/7 sequences may fail to form mature virus particles (VP) or assembly intermediates without a comparable reduction in late protein synthesis^36^. E4orf6/7 may be necessary for capsid assembly and packaging, suggesting that E4 34K may play an important role in capsid assembly. We have observed that the amino acid stretch between residues 192 and 196 with a sequence of WYDGH is conserved among E4 34K proteins of 65 human and animal AdVs (Fig. S1). This sequence falls in the region that would correspond to the start of the tunnel loop and also contains negatively charged aspartate at the putative tunnel loop.

### E4 34K is present in the empty and mature virions, interacts with viral packaging machinery, and is present at a single site on the virus surface

**Figure 2:**
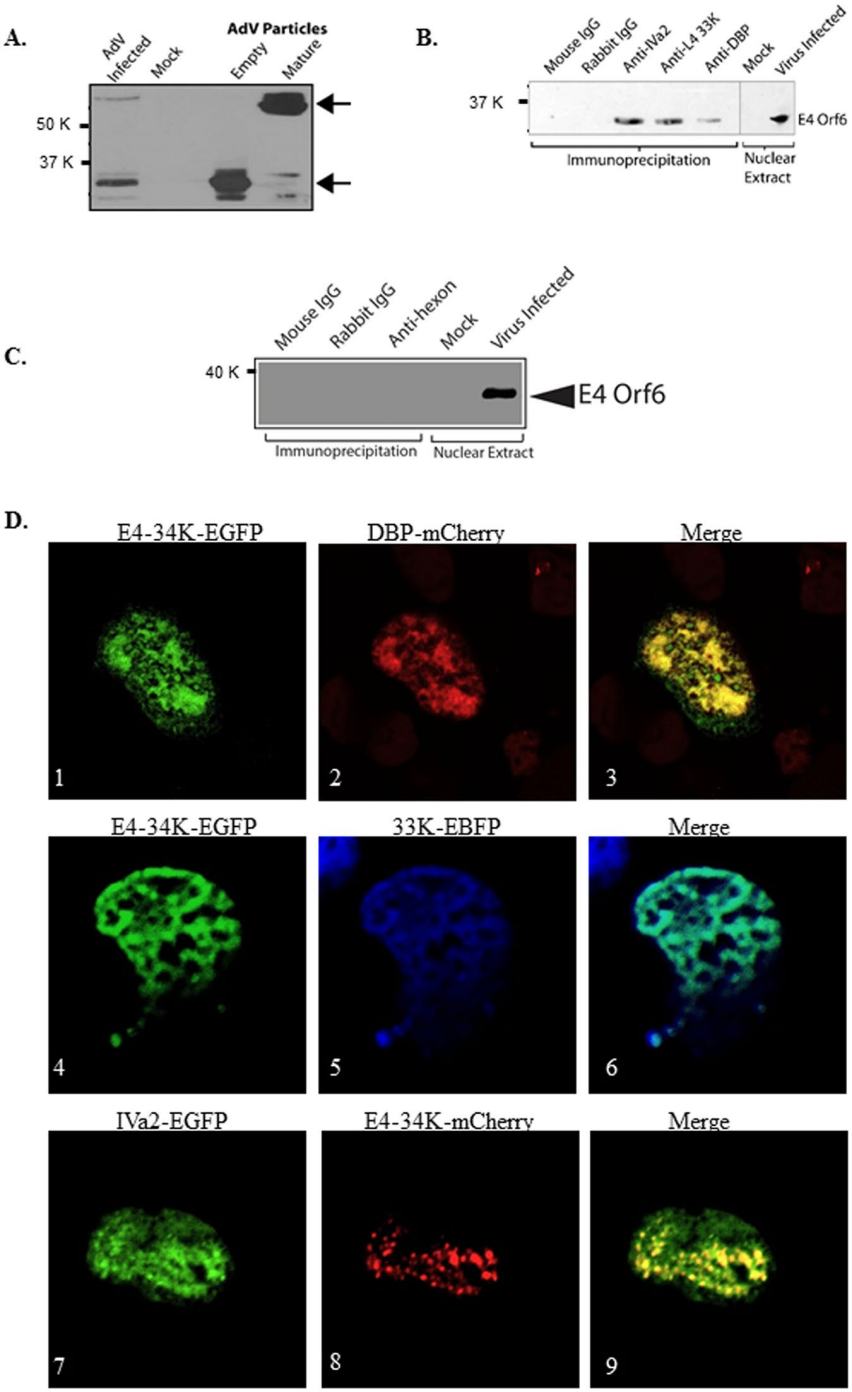
Interaction of E4 34K with AdV packaging proteins. (**A**) Presence of E4 34K in empty and mature AdV particles. Lysates of 10 µg of purified empty or mature particles of HAdV-C5 were separated by SDS–PAGE for immunoblot with anti-E4 34K antibody. The bands corresponding to monomer and dimer of E4 34K are indicated. (**B)** Co-immunoprecipitation of E4 34K with IVa2, 33K and DBP. Nuclear extracts of HAdV-C5 infected cells were immunoprecipitated with anti-IVa2, anti-33K or anti-DBP antibodies, normal mouse IgG or normal rabbit IgG. Immunoprecipitated proteins and total proteins from nuclear extracts of mock-infected and infected cells were separated by SDS-PAGE for immunoblot analysis with anti-E4 34K antibody. (**C)** Nuclear extracts of HAdV-C5 infected cells were immunoprecipitated with anti-hexon antibody, normal mouse IgG or normal rabbit IgG. Immunoprecipitated proteins and total proteins from nuclear extracts of mock-infected and infected cells were separated by SDS-PAGE for immunoblot analysis with anti-E4 34K antibody. (**D)** Co-localization of E4 34K with IVa2, 33K and DBP in 293 cells. 293 cells were transfected with plasmids expressing E4 34K-EGFP and DBP-mCherry (panels 1–3), E4 34K-EGFP and 33K-EBFP (panels 4–6) or IVa2-EGFP and E4 34K-mCherry (panels 7–9) fusion proteins. Cells were analyzed by confocal microscopy 36 h post-transfection. The images were taken at 60× magnification. The images are shown after 5× zoom.

We purified empty and mature HAdV-C5 particles by cesium chloride density gradient centrifugation as described earlier^10^. The densities of empty and mature AdV particles were established to be 1.29–1.30 g/cm^3^ and 1.34 g/cm^3^, respectively. The virus particles to plaque forming units (p.f.u.) ratio of the empty and mature particles was determined to be approximately 10^7^ to 10^1^, respectively, suggesting that the fraction with empty particles was not significantly contaminated with mature particles. Uninfected (mock) or HAdV-C5-infected 293 cells were harvested at 36 h post-infection to serve as negative and positive controls, respectively. The extracts of the mock-infected and AdV-infected cells, as well as purified preparations of empty and mature virus particles were separated by SDS–PAGE for immunoblot analysis with anti-E4 34K antibody. In HAdV-C5-infected cells, E4 34K was detected primarily as a monomer; however, a faint band was also seen running above the 50 kDa MW marker (Fig. 2A). This band could possibly correspond to a dimer of the E4 34K protein. Under denaturing conditions, E4 34K was observed predominantly as a monomer in the empty capsids, whereas in the mature particles, the majority of E4 34K was visualized in the form of a dimer (Fig. 2A). This experiment was repeated three times with different virus preparations, and similar results were obtained. The samples were heated at 50 °C before loading onto SDS-PAGE gels as the anti-E4 34K antibody works better under these conditions. Heating at 50 °C is anticipated to cause incomplete denaturation (rather than complete denaturation at boiling temperature). Partial denaturation at 50 °C is one of the possible reasons for the appearance of E4 34K predominantly as a dimer in the mature particles.

The next criterion that the AdV portal must satisfy is to interact with the viral packaging machinery including the packaging ATPase (also known as larger terminase) and the small terminase. IVa2 seems to act as the packaging ATPase, whereas the L4 33K (will be referred to as 33K for simplicity) possibly functions as small terminase equivalent and potentiates ATPase activity of IVa2^9^. The AdV DBP interacts with IVa2 and 33K, and is located at a unique vertex at AdV capsids^10, 13^. Given these observations, it is assumed that the AdV portal will form a complex with IVa2, 33K and DBP. In order to test this hypothesis, we performed immunoprecipitation of nuclear extracts of HAdV-C5-infected 293 cells with anti-IVa2, anti-33K, anti-DBP or anti-hexon antibodies using a protocol as described earlier^10^. The immunoprecipitations were performed with the nuclear extracts since AdV capsid assembly and genome packaging are expected to occur in the nucleus. Immunoprecipitates were separated by SDS–PAGE for immunoblot analysis with anti-E4 34K antibody. As anticipated, E4 34K was detected in the protein complexes immunoprecipitated from the nuclear extracts of HAdV-C5-infected cells with anti-IVa2, anti-33K and anti-DBP antibodies, but not with the mouse IgG or rabbit IgG (Fig. 2B) or with anti-hexon antibody (Fig. 2C).

To further validate the above results, we performed confocal microscopy to visualize co-localization of E4 34K with IVa2, 33K and DBP. 293 cells were transfected with various combinations of plasmids pEGFP/IVa2, pEBFP/33K, pmCherry/DBP, pEGFP/E4-34K and pmCherry/E4-34K. Specifically, the fusion proteins were expressed in the following combinations: E4 34K-EGFP and DBP-mCherry, E4 34K-EGFP and 33K-EBFP or IVa2-EGFP and E4 34K-mCherry. The transfected cells were examined by confocal microscopy at 36 h post-transfection. Superimposition of the images from green (E4 34K-EGFP) & red (DBP-mCherry), green (E4 34K-EGFP) & blue (33K-EBFP), and green (IVa2-EGFP) & red (E4 34K-mCherry) channels resulted in yellow, cyan and yellow fluorescence, respectively (Fig. 2D). These results are consistent with colocalization, even in the absence of other viral proteins and the viral genome. The results of confocal microscopy, in combination with the results of immunoprecipitation experiments, strongly suggest that E4 34K interacts with the proteins of AdV packaging machinery.

**Figure 3:**
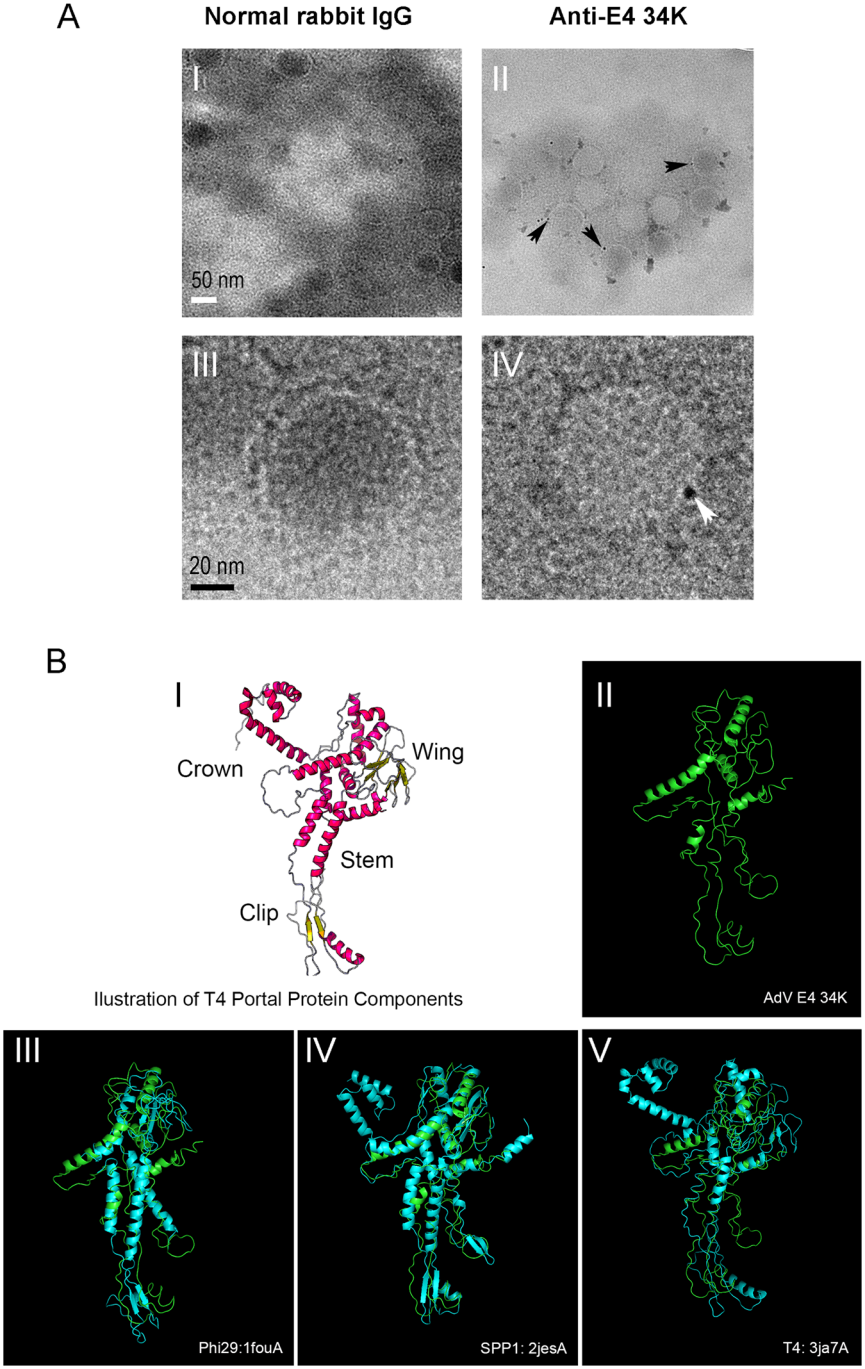
**(A)** Immunogold EM of HAdV-C5 particles labeled with anti-E4 34K antibody. A purified HAdV-C5 preparation was applied to the grids and treated with anti-E4 34K antibody or normal rabbit IgG followed by treatment with anti-rabbit antibody conjugated to 6 nm gold particles. The grids were examined by transmission electron microscope (TEM) with a Philips CM200FEG electron microscope. The low magnification photos were taken at 13.5K and the high magnification photos were taken at 58K using a Gatan Ultrascan4000 CCD camera. (**B**) Predicated tertiary structure of AdV E4 34K. I Predicted tertiary structure of a single subunit T4 portal protein by RaptorX^48^, which is close to the recently published T4 portal structure^31, 34, 35^, suggesting that the predicted structure is identical in most parts with the experimental one. A typical viral portal protein contains a crown, wing stem and clip^31, 34, 35^. II Predicted tertiary structure of AdV E4 34K by I-TSAASER using template guiding model. III–V E4 34K (green) is superimposed with a single subunit of SPP1 (2jesA, TM-score = 0.637), T4 (3ja7A, TM-score = 0.513), and Phi29 (1fouA, TM-score = 0.391) portal proteins^31, 35, 49^.

One of the key features of a portal is its presence only at a unique vertex on the virus surface. To examine the distribution of E3 34K on the virion surface, a purified preparation of HAdV-C5 was analyzed by immunogold EM using a protocol described previously^10, 16–25^. Since the portal is expected to be presented in both the empty and mature particles, it was not necessary to separate them for the detection of E4-34K by immunogold EM. Purified HAdV-C5 particles were layered onto Formvar-carbon-coated nickel EM grids and treated with anti-E4 34K antibody or normal rabbit IgG. Specific binding of the primary antibody was detected by anti-rabbit antibody conjugated to 6 nm gold particles. Grids were negatively stained with phosphotungstic acid (PTA) and examined by transmission EM (TEM). The anti-E4 34K antibody-treated virions demonstrated the presence of gold particles at a single location (Fig. 3A, panels II & IV) and immunogold labeling was observed in approximately 16% (8/50 per grid) of AdV particles. Gold particles were observed on both stain-penetrated (empty) and non-penetrated (mature) particles. This observation was anticipated since E4 34K is present in both the empty and mature particles. Immunogold labeling with anti-HAdV-C5 hyperimmune serum resulted in the labeling of capsids at more than one location (data not shown). No signal was detected with a normal rabbit IgG (Fig. 3A, panels I & III)). To rule out the possibility of non-specific binding of the anti-E4 34K antibody, the immunogold EM protocol was repeated with a purified preparation of T7 bacteriophage and no labeling was detected (data not shown). This result shows the presence of E4-34K at a single site on the AdV surface. In our earlier study, the components of the AdV packaging complex including IVa2, 33K and DBP were detected by immunogold EM labeling of approximately 22, 11 and 15% of AdV particles, respectively^10, 16–25^.

### Predicted tertiary structure of E4 33K and its comparison with the known tertiary structure of single subunit of Phi29, SPP1 and T4 portal proteins

The protein tertiary structure is critical for understanding of the function of a given protein. Tertiary structures of portal proteins of some bacteriophages have been deciphered. We reasoned that if E4 34K serves as a portal protein for AdV, its tertiary structure may have some similarities with the known portal proteins. Since getting the crystal structure will take enormous time and effort, we decided to take the protein prediction approach for this purpose. Though the tertiary structure of E4 34K was previously predicted using the fold recognition method^37^, the accuracy of the prediction is not known due to the unavailability of the experimental structures in the PDB databases. Recently, structurally matching the 3D models with other known proteins was found to be more reliable^38, 39^, since many structures of portal proteins were deposited in the PDB. As a result, we decided to predict the tertiary structure using the recent state-of-art technology. We successfully predicted the tertiary structure of E4 34K (Fig. 3B, panel II) by I-TASSER, one of the most commonly used programs^40^. The predicted structure of E4 34K aligned well with the other portal proteins of Phi29, SPP1 and T4 (Fig. 3B, panels III, IV & V). More importantly, the predicted “clip region” of E4 34K closely matched both the secondary **(**Fig. 1 **)** and tertiary structures **(**Fig. 3B, panels III, IV & V) of the Phi29, SPP1 and T4 portals, suggesting that E4 34K is a putative AdV portal protein.

In conclusion, a combination of biochemical and protein structure prediction analyses provide reasonable evidence suggesting that E4 34K is a putative AdV portal protein. Nevertheless, additional studies including the characterization of E4 34K mutants and the detailed 3D structure analysis of E4 34K will provide the needed evidence to confirm the findings described in this manuscript.

## Materials and Methods

### Cell lines, viruses and plasmids

HEK 293 (p31), a human embryonic kidney cell line transformed with E1 region of HAdV-C5^41^, was obtained from ATCC. 293cre cell line (p10), a derivative of HEK 293 cells that constitutively expresses Cre recombinase^42^, was obtained from Dr. Frank Graham, McMaster University, Hamilton, Ontario, Canada and Dr. Andy Bett, Merck, Kenilworth, NJ, United States. These cell lines were maintained in minimum essential medium (MEM) with 10% FetalClone III serum (Thermo Scientific, Rockford, IL). Viruses used in this study were HAdV-C5 and ADLC8cluc, an HAdV-C5 vector in which the packaging signal is flanked by *loxP* sites^42^. HAdV-C5 was obtained from ATCC. ADLC8cluc was obtained from McMaster University, Hamilton, Ontario, Canada and Merck, Kenilworth, NJ, United States. The HAdV-C5 and ADLC8cluc were grown in 293 or 293cre, respectively to purify the mature or empty virion particles using a continuous cesium chloride density gradient and ultra-centrifugation as described earlier^43^. Virus titers were determined by plaque assay on BHH2C (bovine-human hybrid clone 2 C) cells^44^. Purification of empty and mature virus particles was performed as described earlier^45^. Plasmids pEGFP/IVa2, pEBFP/33K and pmCherry/DBP have been described earlier^10^. Plasmid pEGFP/E4-34K is pcDNA3.1^+^ expressing E4 34K-EGFP fusion protein (EGFP fused at the N-terminal end of E4 34K). Plasmid pmCherry/E4-34K is pcDNA3.1^+^ expressing E4 34K-mCherry fusion protein (mCherry fused at the C-terminal end of E4 34K).

### Antibodies and immunoblotting

Anti-E4 34K antibody was kindly provided by Dr. Philip Branton (McGill University, Montreal, Québec, Canada). Anti-IVa2 and anti-33K antibodies have been described earlier^10^. Anti-DBP antibody clone B6^46^ was kindly provided by Dr. Arnold Levine (Institute of Advanced Study, Princeton, NJ). Cells extracts or purified virus particles were processed for immunoblotting as described earlier^44^. The HAdV-C5 hexon mouse monoclonal antibody (clone: 65H6) was purchased from ThermoFisher Scientific and it recognizes a linear epitope.

### Co-Immunoprecipitation

Preparation of nuclear extracts from HAdV-C5-infected cells and co-immunoprecipitation with anti-IVa2, anti-33K and anti-DBP antibodies from nuclear extracts have been described earlier^10^. The immunoprecipitated complexes were analyzed by SDS-PAGE followed by immunoblotting with anti-E4 34K antibody.

### Confocal microscopy

293 cells were grown on coverglasses (Corning, Corning, NY) pre-coated with poly-l-lysine. At about 60% confluency, cells were transfected with plasmids pEGFP-IVa2, pEBFP-33K, pmCherry-DBP, pEGFP/E4-34K and pmCherry/E4-34K in various combinations. The transfections were done using Lipofectamine 2000 following manufacturer’s instructions. At 36 h post-transfection, the cells were washed once with PBS followed by fixation with 4% PFA in PBS for 15 min at room temperature. The coverglasses were mounted on glass slides using Fluoro-gel mounting medium (Electron Microscopy Sciences, Hatfield, PA). Fluorescence imaging was performed using a Nikon C1+ confocal microscope (Nikon, Melville, NY) with 60× objective. Image analyses were performed using NIS Elements software (Nikon).

### Immunogold electron microscopy

Purified preparations of HAdV-C5 were used for immunogold electron microscopy, as described elsewhere^10^, with anti-E4 34K antibody as primary antibody and anti-rabbit antibody labeled with 6 nm gold particles as secondary antibody.

### Multiple protein sequence alignment, and prediction of secondary and tertiary structures

E4 34K homologous proteins in 65 different human and animal AdVs were identified using NCBI BLASTp. Sequences of these proteins were aligned with E4 34K of HAdV-C5 using COBALT (Constraint Based Multiple Alignment Tool) at the NCBI website. Protein sequences of E4 34K and other portal proteins were retrieved from the NCBI website: E4 34K (AP_000228.1), phage phi29 (P04332.1), phage portal protein, SPP1 (GAP04951.1), and bacteriophage T4 Portal Protein (3JA7_L). Sequences were submitted to PSIPRED and I-TASSER through their online websites for secondary and tertiary structure predictions, respectively^40, 47^. Consensus secondary structures were extracted from both prediction results. For tertiary structure prediction, protein sequences were analyzed using RaptorX Structure Prediction ^48^ and I-TASSER^40^ guiding model (Phi29 was used as a template) was employed using their online programs with default settings. The final structure images were generated with MacPyMOL (v1.8.4.0).

## Electronic supplementary material

Figure S1
