# Supplementary material for: Adenoviral E4 34K protein interacts with virus packaging components and may serve as the putative portal
Source: Sci Rep. 2017 Aug 8;7:7582. doi: 10.1038/s41598-017-07997-w (PMC5548797; doi:10.1038/s41598-017-07997-w)
Supplement: Supplementary file 1 — Figure S1 [file 41598_2017_7997_MOESM1_ESM.doc]

# Adenoviral E4 34K protein interacts with virus packaging components and may serve as the putative portal

Yadvinder S. Ahi^1,4,5,§^ , Ahmed O. Hassan^1,3,4, §^, Sai V. Vemula^1,4,6^, Kunpeng Li^2,3^, Wen Jiang^,2,3^, GuangJun Zhang^1,3,4^ and Suresh K. Mittal^1,3,4,*^

^1^Department of Comparative Pathobiology, ^2^Department of Biological Sciences, ^3^Purdue Institute of Inflammation, Immunology, and Infectious Disease, and ^4^Purdue University Center for Cancer Research, Purdue University, West Lafayette, IN, USA

**^5^**Present Address: HIV Dynamics and Replication Program, Center for Cancer Research, National Cancer Institute, Frederick, MD, USA

^6^Merck Sharp and Dohme, West Point, PA, USA

§: These authors have equal contribution.

***Correspondence**: Suresh K. Mittal, Department of Comparative Pathobiology, College of Veterinary Medicine, Purdue University, 725 Harrison St. West Lafayette, IN 47907-2027, USA

Email: [mittal@purdue.edu](mailto:mittal@purdue.edu)

**Supplementary Fig. S1. Conservation of the clip region of E4 34K in various AdVs.** The amino acid motif (WYDGH) at the tunnel entrance is indicated by a line on the top of the residues. Red residues: conserved/cannot be substituted; Blue residues: somewhat conserved/can be substituted.

**Tunnel entrance**

Query_187004 159 IWYREVVNYNMPKEVMFMSSVFMRGRHLIYLRLWYDGHVGSVVPAMSFGYSALHCGILNNIVVLCCSYCADLSEIRVRCC 238

AFS50351 159 IWYREVVNYNMPKEVMFMSSVFMRGRHLIYLRLWYDGHVGSVVPAMSFGYSALHCGILNNIVVLCCSYCANLSEIRVRCC 238

AFS50440 159 IWYREVVNYNMPKEVMFMSSVFMRGRHLIYLRLWYDGHVGSVVPAMSFGYSALHCGILNNIVVLCCSYCADLSEIRVRCC 238

AGV40532 159 LWYREVVNQHMPKEVMYMSSVFMRGRHLIYLRIWYDGHVGAVLPAMSFGYSALHCGILNNILVLCCTYCADLSEIRMRCC 238

AGV40533 159 LWYREVVNQHMPKEVMYMSSVFMRGRHLIYLRIWYDGHVGAVLPAMSFGYSALHCGILNNILVLCCTYCADLSEIRMRCC 238

AGV40531 159 LWYREVVNQRMPKEVMYMSSVFMRGRHLIYLRIWYDGHVGAVLPAMSFGYSALHCGILNNILVLCCTYCADLSEIRMRCC 238

AFK92674 149 PWYRQIVNRDMPKEIMYMGSVFMRGRHLIYCRIWYDGHVGSIIPNMSFGWSALNYGLLNNMVIMCCTYCENLSEIRMRCC 228

AFK92834 149 PWYRQIVNRNMPKEIMYMGSVFMRGRHLIYCRIWYDGHVGSIIPNMSFGWSTLNYGLLNNMVIMCCTYCENMSEIRMRCC 228

AGT76472 149 PWYRQIVNRDMPKEIMYMGSVFMRGRHLIYCRIWYDGHVGSIIPNMSFGWSALNYGLLNNMVIMCCTYCENLSEIRMRCC 228

AFK92754 149 PWYRQIVNKNMPKEIMYMGSVFMRGRHLIYCRIWYDGHVGSIIPNMSFGWSTLNYGLLNNMVIMCCTYCENMAEIRMRCC 228

ABK59081 149 PWYRQIVNRNMPKEIMYMGSVFMRGRHLIYCRIWYDGHVGSIIPNMSFGWSALNYGLLNNMVIMCCTYCENMAEIRMRCC 228

ACY04489 149 PWYRQIVNRNMPKEIMYMGSVFMRGRHLIYCRIWYDGHVGSIIPNMSFGWSTLNYGLLNNMVIMCCTYCENMSEIRMRCC 228

BAH18943 149 PWYRQIVNRNMPKEMMYMGSVFMRGRHLIYCRIWYDGHVGSIIPNMSFGWSALNYGLLNNMVIMCCTYCENMAEIRMRCC 228

ABN10544 149 PWYRQIVNRNMPKEIMYMGSVFMRGRHLIYCRIWYDGHVGSIIPNMSFGWSALNYGLLNNMVIMCCTYCENMAEIRMRCC 228

AEI91306 149 PWYRQIVNRDMPKEIMYMGSVFMRGRHLIYCRIWYDGHVGSIIPNMSFGWSALNYGLLNNMVIMCCTYCENLSEIRMRCC 228

AEL78858 149 PWYRQIVNRNMPKEIMYMGSVFMRGRHLIYCRIWYDGHVGSIIPNMSFGWSALNYGLLNNMVIMCCTYCENMAEIRMRCC 228

AFK92554 149 PWYRQIVNRNMPKEIMYMGSVFMRGRHLIYCRIWYDGHVGSIIPNMSFGWSTLNYGLLNNMVIMCCTYCENMAEIRMRCC 228

BAJ22305 149 PWYRQIVNRDMPKEIMYMGSVFMRGRHLIYCRIWYDGHVGSIIPNMSFGWSALNYGLLNNMVIMCCTYCENMAEIRMRCC 228

AFK92434 149 PWYRQIVNRNMPKEIMYMGSVFMRGRHLIYCRIWYDGHVGSIIPNMSFGWSTLNYGLLNNMVIMCCTYCENMSEIRMRCC 228

AGW47861 149 PWYRQIVNRNMPKEIMYMGSVFMRGRHLIYCRIWYDGHVGSIIPNMSFGWSALNYGLLNNMVIMCCTYCENMAEIRMRCC 228

AFK92714 149 PWYRQIVNRDMPKEIMYMGSVFMRGRHLIYCRIWYDGHVGSIIPNMSFGWSTLNYGLLNNMVIMCCTYCENMSEIRMRCC 228

AFK92634 149 PWYRQIVNRDMPKEIMYMGSVFMRGRHLIYCRIWYDGHVGSIIPNMSFGWSTLNYGLLNNMVIMCCTYCENMAEIRMRCC 228

AFK92794 149 PWYRQIVNRDMPKEIMYMGSVFMRGRHLIYCRIWYDGHVGSIIPNMSFGWSTLNYGLLNNMVIMCCTYCENMAEIRMRCC 228

AFK92594 149 PWYRQIVNRDMPKEIMYMGSVFMRGRHLIYCRIWYDGHVGSIIPNMSFGWSALNYGLLNNMVIMCCTYCENMAEIRMRCC 228

AEY79591 149 PWYRQIVNRDMPKEIMYMGSVFMRGRHLIYCRIWYDGHVGSIIPNMSFGWSTLNYGLLNNMVIMCCTYCENMSEIRMRCC 228

ADM66132 149 PWYRQIVNRNMPKEIMYMGSVFMRGRHLIYCRIWYDGHVGSIIPNMSFGWSTLNYGLLNNMVIMCCTYCENMAEIRMRCC 228

AGT78020 149 PWYRQIVNRDMPKEIMYMGSVFMRGRHLIYCRIWYDGHVGSIIPNMSFGWSTLNYGLLNNMVIMCCTYCENMAEIRMRCC 228

AAS10386 158 MWYREVVNRHMPKEIMYMGSVFWRGRHLIYLRIWYDGHVGSILPAMSFGWSVLNYGLLNNLVVLCCTYCSDLSEIRMRCC 237

BAJ22341 149 PWYRQIVNRDMPKEIMYMGSVFMRGRHLIYCRIWYDGHVGSIIPNMSFGWSTLNYGLLNNMVIMCCTYCENMAEIRMRCC 228

ACR78232 149 PWYRQIVNRDMPKEIMYMGSVFMRGRHLIYCRIWYDGHVGSIIPNMSFGWSTLNYGLLNNMVIMCCTYCENMAEIRMRCC 228

AFK92275 149 PWYRQIVNRDMPKEIMYMGSVFMRGRHLIYCRIWYDGHVGSIIPNMSFGWSTLNYGLLNNMVIMCCTYCENMAEIRMRCC 228

AFK92235 149 PWYRQIVNRDMPKEIMYMGSVFMRGRHLIYCRIWYDGHVGSIIPNMSFGWSTLNYGLLNNMVIMCCTYCENMSEIRMRCC 228

AFK92354 149 PWYRQIVNRDMPKEIMYMGSVFMRGRHLIYCRIWYDGHVGSIIPNMSFGWSTLNYGLLNNMVIMCCTYCENMSEIRMRCC 228

YP_003038627 149 PWYRQIVNRNMPKEIMYMGSVFIRGRHLIYCRIWYDGHVGSIIPNISFGWSTLNYGLLNNIMIMCCTYCENMSEIRMRCC 228

AEK87042 149 PWYRQIVNRDMPKEIMYMGSVFMRGRHLIYCRIWYDGHVGSIIPNMSFGWSTLNYGLLNNMVIMCCTYCENMAEIRMRCC 228

AFK92514 149 PWYRQIVNRDMPKEIMYMGSVFMRGRHLIYCRIWYDGHVGSIIPNMSFGWSALNYGLLNNMVIMCCTYCENMAEIRMRCC 228

AFK92195 149 PWYRQIVNRDMPKEIMYMGSVFMRGRHLIYCRIWYDGHVGSIIPNMSFGWSTLNYGLLNNMVIMCCTYCENMAEIRMRCC 228

AGT77183 149 PWYRQIVNRDMPKEIMYMGSVFMRGRHLIYCRIWYDGHVGSIIPNMSFGWSALNYGLLNNMVIMCCTYCENMAEIRMRCC 228

BAH18835 149 PWYRQIVNRNMSKEIMYMGSVFIRGRHLIYCRIWYDGHVGSIISNMSFGWSTLNYGLLNNMVIMCCTYCENMSEIRMQCC 228

BAH18799 149 PWYRQIVNRNMSKEIMYMGSVFIRGRHLIYCRIWYDGHVGSIISNISFGWSTLNYGLLNNMVIMCCTYCENMSEIRMRCC 228

ACI04199 149 PWYRQIVNRNMSKEIMYMGSVFIRGRHLIYCRIWYDGHVGSIISNISFGWSTLNYGLLNNMVIMCCTYCENMSEIRMRCC 228

BAH18871 149 PWYRQIVNRNLSKEIMYMGSVFIRGRHLIYCRIWYDGHVGSIISNISFGWSTLNYGLLNNMVIMCCTYCENMSEIRMRCC 228

YP_068050 159 MWYREVVNLHMPKEIMYMGSVFWRGRHLIYIRIWYDGHVGSIVPQMSFGWSTLNYGLLNNLVVLCCTYCSDLSEIRIRCC 238

AP_000603 159 PWYRQIVNMHMPKEIMYVGSVFLRERHLIYIKLWYDGHAGAIISDMSFGWSAFNYGLLNNIVIMCCTYCKDLSEIRMRCC 238

AIF29743 159 PWYRQIVNMHMPKEIMYVGSVFLRERHLIYIKLWYDGHAGAIISDMSFGWSAFNYGLLNNIVIMCCTYCSNLSEIRMRCC 238

YP_006272975 159 MWYREVVNLHMPKEIMYMGSVFWRGRHLIYIRIWYDGHVGSIVPQMSFGWSTLNYGLLNNLVVLCCTYCSDLSEIRIRCC 238

AET87290 159 PWYRQIVNMHMPKEIMYVGSVFLRERHLIYIKLWYDGHAGAIISDMSFGWSAFNYGLLNNIVIMCCTYCSNLSEIRMRCC 238

AAZ32190 159 PWYRQIVNMHMPKEIMYVGSVFLRERHLIYIKLWYDGHAGAIISDMSFGWSAFNYGLLNNIVIMCCTYCSNLSEIRMRCC 238

AAW33549 159 PWYRQIVNMHMPKEIMYVGSVFLRERHLIYIKIWYDGHAGAIVSDMSFGWSAFNYGLLNNIVIMCCTYCSNLSEIRMRCC 238

ACX32426 159 PWYRQIVNMHMPKEIMYVGSVFLRERHLIYIKIWYDGHAGAIISDMSFGWSAFNYGLLNNIVIMCCTYCSNLSEIRMRCC 238

AET87167 159 PWYRQIVNMHMPKEIMYVGSVFLRERHLIYIKIWYDGHAGAIISDMSFGWSAFNYGLLNNIVIMCCTYCSNLSEIRMRCC 238

AAY21870 159 PWYRQIVNMHMPKEIMYVGSVFLRERHLIYIKIWYDGHAGAIISDMSFGWSAFNYGLLNNIVIMCCTYCSNLSEIRMRCC 238

AAY21874 153 PWYRQIVNMHMPKEIMYVGSVFLRERHLIYIKLWYDGHAGAIISDMSFGWSAFNYGLLNNIVIMCCTYCSNLSEIRMRCC 232

AAW33278 159 PWYRQIVNMHMPKEIMYVGSVFLRERHLIYIKLWYDGHAGAIISDMSFGWSAFNYGLLNNIVIMCCTYCSNLSEIRMRCC 238

AFV96309 159 PWYRQIVNMHMPKEIMYVGSVFLRERHLIYIKIWYDGHAGAIISDMSFGWSAFNYGLLNNIVIMCCTYCSNLSEIRMRCC 238

AAW33419 159 PWYRQIVNMHMPKEIMYVGSVFLRERHLIYIKIWYDGHAGAIISDMSFGWSAFNYGLLNNIVIMCCTYCSNLSEIRMRCC 238

AP_000566 159 PWYRQIVNMHMPKEIMYVGSVFLRERHLIYIKIWYDGHAGAIISDMSFGWSAFNYGLLNNIVIMCCTYCSNLSEIRMRCC 238

AAT97565 159 PWYRQIVNMHMPKEIMYVGSVFLRERHLIYIKIWYDGHAGAIISDMSFGWSAFNYGLLNNIVIMCCTYCSNLSEIRMRCC 238

AAY21878 153 PWYRQIVNMHMPKEIMYVGSVFLRERHLIYIKIWYDGHAGAIISDMSFGWSAFNYGLLNNIVIMCCTYCSNLSEIRMRCC 232

AFI24604 159 PWYRQIVNMHMPKEIMYVGSVFLRERHLIYIKIWYDGHAGAIISDMSFGWSAFNYGLLNNIVIMCCTYCSNLSEIRMRCC 238

AAW33234 159 MWYREVVNLHMPKEIMYMGSVFWRGRHLIYIRIWYDGHVGSIVPQMSFGWSTLNYGLLNNVVVLCCTYCFDLSEIRIRCC 238

ACU57031 159 PWYRQIVNMHMPKEIMYVGSVFLRERHLIYIKLWYDGHAGAIISDMSFGWSAFNYGLLNNIVIMCCTYCINLSEIRMRCC 238

AAW33142 159 PWYRQIVNMHMPKEIMYVGSVFLRERHLIYIKLWYDGHAGAIISDMSFGWSAFNYGLLNNIVIMCCTYCINLSEIRMRCC 238

ACO81815 152 PWYRQIVNMHMPKEIMYVGSVFLRERHLIYIKLWYDGHAGAIISDMSFGWSAFNYGLLNNIVIMCCTYCINLSEIRMRCC 231

AHJ81847 159 PWYRQIVNMHMPKEIMYVGSVFLRERHLIYIKIWYDGHAGAIISDMSFGWSAFNYGLLNNIVIMCCTYCSNLSEIRMRCC 238

AET87331 159 PWYREIVNMHMPKEIMYVGSVFWRGRHLIYIKIWYDGHAGMIIPEMSFGWSTFNYGLLNNIVVMCCTYCSELSEIRMRCC 238
